# Histological endometrial dating: a reliable tool for personalized frozen-thawed embryo transfer in patients with repeated implantation failure in natural cycles

**DOI:** 10.1186/s12884-023-05512-w

**Published:** 2023-03-22

**Authors:** Yuan Li, Quan Wen, Jing Hu, Jingnan Liao, Xiangxiu Fan, Huijun Chen, Qi Zhao, Guang-Xiu Lu, Ge Lin, Fei Gong

**Affiliations:** 1 Reproductive and Genetic Hospital of CITIC-Xiangya, Changsha, China; 2 NHC Key Laboratory of Human Reproductive and Stem Cell Engineering, Changsha, China; 3 Institute of Reproductive and Stem Cell Engineering, School of Basic Medical Science, Central South University, Changsha, China

**Keywords:** Histological endometrial dating, Repeated implantation failure, Personal embryo transfer, Window of implantation

## Abstract

**Objective:**

To evaluate the clinical availability and stability of histological endometrial dating as a tool for personalized frozen-thawed embryo transfer (pFET) in patients with repeated implantation failure (RIF) in natural cycles.

**Methods:**

A total of 1245 RIF patients were recruited to the present study. All of the patients received an endometrial dating evaluation on day 7 post-ovulation (PO + 7) to guide their first pFET. The second and third pFETs were executed according to histological examination (again employing biopsy) or by reference to previous results. Subsequent pregnancy outcomes for all of the cycles were ultimately tracked.

**Results:**

The out-of-phase rate for RIF patients was 32.4% (404/1245) and the expected dating rate (the probability of the expected endometrial dating aligning with repeat biopsy) for endometrial dating reevaluation was as high as 94.3% (50/53). The clinical pregnancy rates of first, second, and third pFETs were 65.3%, 50.0%, and 44.4%, respectively; and the cumulative clinical pregnancy rate attained 74.9% after three transfers. Endometrial dating reevaluations met expectations with more than a 2-year duration in three cases and elicited favorable clinical outcomes.

**Conclusion:**

We validated the relatively high stability of the histological endometrial dating platform—including the out-of-phase rate and the expected dating rate of reevaluation in patients with RIF—by expanding the sample size. The pFET, based on histological endometrial dating, was of acceptable clinical value and was worthy of promotion in patients with unexplained RIF.

## Introduction

Repeated implantation failure (RIF) is a form of refractory infertility that refers to the absence of a gestational sac at 5 or more weeks; this is followed by embryo transfer (ET)—with three transfers of high-quality embryos or after the transfer of ≥ 10 embryos in multiple transfers [1]. The etiology of RIF can be attributed to the embryonic quality, endometrial dysfunction, uterine anomaly, immunological factors, thrombophilia, hormonal or metabolic disorders, and/or genetic predisposition [2–5]. However, the cause of RIF remains obscure in most cases, and unexplained RIF has become a frequently encountered and difficult clinical challenge [6].

Insufficient endometrial receptivity leading to altered embryo-endometrial synchrony is a leading cause of RIF [7]. Endometrial receptivity is defined as a transient period in which the endometrium is prepared to receive the implanting embryo, and is also known as the “window of implantation” (WOI)[8]. The characteristics of the WOI have achieved a favored status in reproductive research over many decades, and relevant assessments of the WOI include ultrasonographic imaging, endometrial biopsy, endometrial-fluid aspirate, or hysteroscopy, but little progress has been made on clinically meaningful prognostic tests and treatments [9].

In the 1950s, Noyes et al. created the classical histological criteria used to evaluate the endometrium on each specific day after ovulation, based on morphological changes of the epithelium and stroma [10]. According to the Noyes criteria, histological dating of a timed endometrial biopsy specimen is made available to assess the WOI, and then embryonic transfer time is adjusted in a subsequent cycle to restore the synchrony between embryonic and endometrial development [11]. However, the subjective nature of such assessments [12] and the apparently substantial variation in results of different studies [13–15] have greatly limited the clinical application of such histological dating.

In our previous study, we first verified the Noyes criteria in pregnant patients [16], and “out-of-phase” designated a difference of more than 2 days between the histological date and the actual day after ovulation [17]. We then executed personalized frozen-thawed embryo transfer (pFET) in the RIF patients that were “out-of-phase”, and found that histological endometrial dating improved the clinical outcomes of those patients with unexplained RIF. However, our results were limited by a small sample size, which may have biased our conclusions. The clinical feasibility, reliability, and repeatability of histological endometrial dating for RIF patients would thus require the clinical validation of a larger sample size.

## Materials and methods

### Study population

**Fig. 1:**
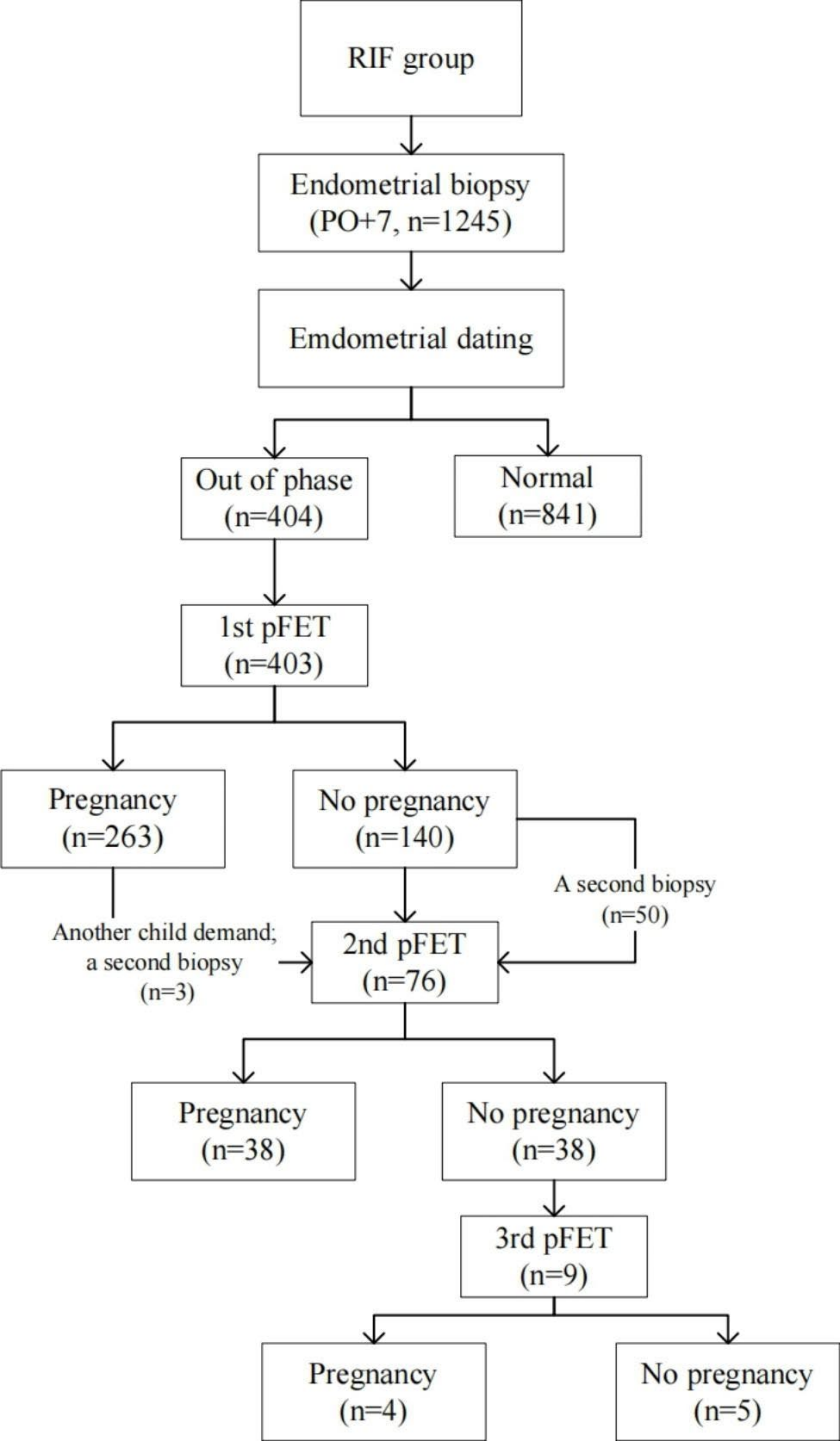
Patients included in the study

This was a retrospective cohort study. A total of 1245 patients with unexplained RIF were recruited for endometrial dating evaluation on day 7 post-ovulation (PO + 7) from January 2018 to December 2020 in the Reproductive and Genetic Hospital of CITIC-Xiangya. The patients regarded as out-of-phase received pFET one to three times; the second and third embryo transfers were based on endometrial dating by a repeated biopsy or with reference to previous results (additional details are shown in Fig. 1). RIF was determined by the absence of a gestational sac at 5 or more weeks followed by embryo transfer (ET) that involved three transfers of high-quality embryos or after the transfer of ≥ 10 embryos in multiple transfers [1]. **As we described previously**[16], we **included** patients with a menstrual-cycle duration ranging from 24 to 35 days, and who were referred for ovarian stimulation before in vitro fertilization/intracytoplasmic sperm injection (IVF/ICSI). Patients with the following reproductive anomalies were excluded: anatomic abnormalities of the uterus, intrauterine adhesions, endometriosis, adenomyosis, hydrosalpinx, or uterine fibroids. Ethical approval for this study was obtained from the Ethics Committee of CITIC-Xiangya, the People’s Republic of China (LL-SC-2022-007).

### Clinical procedures

Procedures were performed per our previous descriptions [16]. We conducted ovulation monitoring and designated the day of dominant follicle disappearance as the day of ovulation. Tissue sections from endometrial biopsy were then stained with hematoxylin and eosin (H&E) and subsequently used for histological endometrial dating. We transferred a maximum of two embryos per patient in the frozen-thawed embryo transfer (FET) cycle, and according to the endometrial dating results, patients underwent FET on the days 4–7 after ovulation, and their pregnancy outcomes were noted. Clinical pregnancy was defined as a pregnancy with at least one intrauterine gestational sac on day 28 after ET, and implantation rate was defined as the percentage of transferred embryos that successfully underwent implantation vs. all transferred.

### Statistical analysis

We executed all statistical analyses using SPSS 26.0 (IBM). Continuous data are presented as means and standard deviation **or median (interquartile range), where applicable**. Categorical data are presented as counts (percentages) and analyzed with the Chi-squared test.

## Results

### Out-of-phase rate in RIF patients

Histological endometrial dating in the first evaluation revealed an out-of-phase rate in RIF patients of 32.4% (404/1245): 6% (n = 23) were dated + 3, 94% (n = 381) were dated + 4 or + 5 (vacuoles remained), and no patient was diagnosed dating + 9 or later. Some patients who failed to become pregnant in their first pFET attempt (n = 50) or sought to have another child after a longer interval (n = 3) were evaluated for endometrial dating again, and the expected dating rate (the probability of meeting the expected endometrial date by performing repeat biopsy) of reevaluation was up to 94.3% (50/53).

### Demographics of out-of-phase RIF patients and clinical outcomes using pFET

As noted above, 404 RIF patients were determined as being out-of-phase, and their demographics and clinical outcomes with respect to pFET are shown in Table 1. These patients were 33.0 ± 3.9 years old, and their body mass index (BMI) was 21.0 ± 1.9 kg/m^2^; the **median** number of previous failed cycles was **3.0**; their **median** duration of infertility was **5.0** years; endometrial thickness on the day of embryo transfer was 11.9 ± 1.4 mm. A total of 403 patients underwent their first pFET with a high-quality embryo rate of 57.3%, and this resulted in an implantation rate of 53.9% and a clinical pregnancy rate of 65.3%. A total of 76 patients accepted a second pFET attempt, including those who failed to become pregnant in the first pFET or sought to have another child. The rate of high-quality embryos transferred was 55.3% in the second pFET, and the implantation and clinical pregnancy rates achieved were 40.4% and 50.0%, respectively. Only nine patients underwent a third pFET cycle, and these showed a high-quality embryo rate of 66.7%, implantation rate of 50.0%, and clinical pregnancy rate of 44.4%. Finally, the cumulative clinical pregnancy rate reached 74.9% (302/403) after three attempts at pFET.

**Table 1:** Demographics of out-of-phase RIF patients and clinical outcomes using pFET.

Characteristic	Out-of-phase RIF (n = 404)
Age (y)	33.0 ± 3.9
BMI (kg/m2)	21.0 ± 1.9
Duration of infertility (year)	5.8 ± 2.7
No. of previously failed cycles	3.6 ± 0.9
Endometrial thickness on the day of embryo transfer (mm)	11.9 ± 1.4
Distribution of out-of-phase dating	
Delayed by 2 days	23
Delayed by 1 day	381
No. of patients with 1st pFET	403
High-quality embryo rate of 1st pFET	57.3% (231/403)
Implantation rate of 1st pFET	53.9% (304/564)
Clinical pregnancy rate of 1st pFET	65.3% (263/403)
No. of patients with 2nd pFET	76
No. of 2nd biopsies on the specified day	53
2nd expected endometrial dating	50
High-quality embryo rate of 2nd pFET	55.3%(42/76)
Implantation rate of 2nd pFET	40.4% (42/104)
Clinical pregnancy rate of 2nd pFET	50.0% (38/76)
No. of patients with 3rd pFET	9
High-quality embryo rate of 3rd pFET	66.7% (6/9)
Implantation rate of 3rd pFET	50.0% (6/12)
Clinical pregnancy rate of 3rd pFET	44.4% (4/9)
Cumulative clinical pregnancy rate after pFET	74.9% (302/403)

Note: variables are expressed as means ± standard deviation, median (interquartile range) or counts (percentages)

### Case presentation

**Fig. 2:**
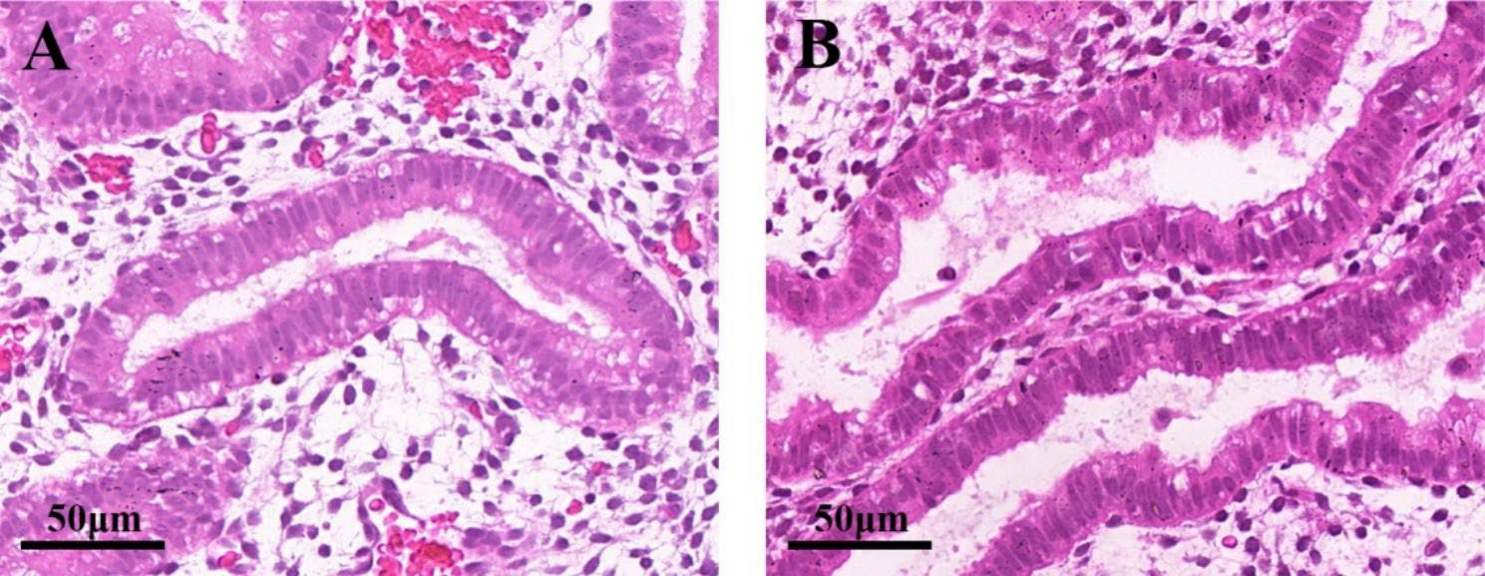
H&E staining of endometrial biopsy tissues on PO + 7. Histological endometrial-dating analysis showed patient endometrium to be dated + 4 in both 2017 (**A**) and 2020 (**B**)

A 33-year-old woman with tubal infertility and a history of four IVF failures with high-quality embryos was referred to our clinic in 2017 to undergo assisted reproductive technology (ART) treatment. Histological endometrial dating results showed that the patient’s endometrium was dated + 4, and an embryo was transferred with a delay of 1 day; the woman then achieved a live birth in the pFET cycle. In May 2020, the patient revisited our clinic seeking a second child and was again ready to accept FET. Since it had been 3 years since the last endometrial dating evaluation, the patient received reevaluation on PO + 7, and results showed the endometrium as dated + 4—the same as observed previously (Fig. 2). Given the clinical outcome of the previous cycle, we advocated the transfer of an embryo with a delay of 1 day, and the patient conceived following the transfer of a grade 4BB blastocyst in July of the same year—recently delivering a healthy baby.

As of now, there were three cases of seeking ART for a second child who underwent pFET 2 years before. Reevaluation of endometrial histological dating in these women was executed prior to FET, and all results met expectations with an even greater than the 2-year duration. Following the same treatment strategy, all three patients achieved clinical pregnancies in their second pFET cycles, resulting in three separate live births.

## Discussion

A successful pregnancy relies primarily upon successful implantation, which is itself a complex reproductive process that involves reciprocal interactions between the endometrium and the embryo [18]. The WOI refers to a narrow time frame of maximal endometrial receptivity, and an endometrial status outside this window is incompatible with pregnancy [9]. The endometrial receptivity array (ERA) test that is based upon a transcriptomic signature has recently emerged as a novel diagnostic tool and has been employed for endometrial dating to guide pFET in RIF patients [19, 20]. The ERA test is, however, in its early stages of exploration, and its extrapolability to the general adapted population remains controversial [21]. In addition, the clinical application of this novel methodology requires support by a corresponding transcriptomics-analysis platform, limiting its promotion and use, and both time and financial costs need to be considered. While the Noyes criteria have been the “gold standard” for endometrial dating since its inception in 1950, some skeptics have recently raised concerns over their accuracy [22, 23]. In our previous pilot study, we verified the endometrial dating criteria in patients with favorable prognoses by performing endometrial biopsies at different time points [16], and we also demonstrated that these criteria were easily mastered and executed with reference to the Noyes criteria.

We had tentatively explored the clinical availability of histological endometrial dating on patients with RIF prior to our pilot study (Study I)[16]. We therein recruited 155 RIF patients: a total of 49 patients were evaluated as being out-of-phase; ultimately, 47 out-of-phase patients underwent pFET once and five did so twice. To verify the reliability and repeatability of histological endometrial dating, we enrolled more than eight times as many patients in the current study (Study II), and results revealed that the out-of-phase rate in RIF patients stabilized at ~ 30% in both our studies. We further reevaluated the endometrial dating of five patients by re-staging the biopsies in Study I, and all five showed the expected endometrial dating results. While the expected dating rate for reevaluation was as high as 94.8% in Study II, the two studies exhibited comparable implantation (47.8% vs. 53.9%, *P* = 0.341) and clinical pregnancy rates (63.8% vs. 65.3%, *P* = 0.846) in the first pFET. Therefore, from the standpoint of WOI asynchrony and the clinical outcomes of pFET, we had reason to believe that the platform of histological endometrial dating was stable with respect to RIF patients.

The clinical pregnancy rate of first, second, and third pFETs in the present study were 65.3%, 50.0%, and 44.4%, respectively; and the cumulative clinical pregnancy rate reached 74.9% after three transfers. The Simón team in Spain reported in a small number of subjects the clinical value of ERA in guiding pFET for RIF patients [19], and they also corroborated this in a large-sample multicenter study in which they targeted a population undergoing IVF with blastocyst transfer at their first appointment [21]. There is currently a lack of other such platforms that employ a large sample size of RIF patients for use in stability verification. In a large retrospective study at our center, the clinical pregnancy rate for the first FET cycle after a previous fresh cycle was 60.25% with thawed cleavage-stage ET and 64.98% with blastocysts cultured from thawed cleavage-stage embryos transferred to young women [24]. Our study revealed that when pFET was applied, the clinical pregnancy rate for RIF patients with asynchrony of their WOI increased to the level of routine patients who underwent FET. Moreover, our data suggested that if there were enough embryos for transfer, the problem of failure to develop to clinical pregnancy could be addressed after 2–3 pFETs in nearly 75% of RIF patients showing WOI displacement.

We observed that the clinical pregnancy rate tended to diminish as the number of pFETs increased. This might have been due to the reduced quality of the transferred embryos in previous pFET cycles, although the number of high-quality embryos transferred was not statistically different. A first pFET may select the highest-quality embryos to be transferred, whereas the quality of the embryos following transplantation may be reduced, even when all of the transferred embryos are of sufficiently high quality. The increasing pressure on patients who experienced several previously failed cycles might be another cause of worse clinical outcomes. The potential causes of RIF include low quality of gametes, and molecular displacements (asynchrony) and molecular disruptions (pathology) within the endometrium [25]. Thus, exploring the pathology of the endometrium before implementing the second/third pFET is of paramount importance. Accordingly, we recommend that other modalities be executed to improve pregnancy outcomes once a patient experiences two pFET failures.

The cases we presented further illustrated that our histological endometrial dating results reflected good reproducibility and verifiability in the same patients, even with a more than the 2-year duration. Histological examination exhibited several other strengths such as simple methodology, low cost, and short reporting periods; and was thus worthy of clinical promotion and application. However, there were some limitations to the present study. Restricted by subjects, we could not design this study as a randomized controlled trial; the results thus still need to be treated with caution. Intriguingly, our data revealed that the WOI for approximately 70% of the RIF patients was not displaced; therefore, the mechanisms underlying implantation failure in this population require further exploration.

In conclusion, by expanding the sample size we further validated the high stability of our histological endometrial dating platform, including the out-of-phase rate and the expected dating rate for the reevaluation of RIF patients. Furthermore, pFET—as an embryo-transfer strategy constructed according to histological endometrial dating— manifested a satisfactory clinical value and was worthy of promotion for patients with unexplained RIF.

## Data Availability

All datasets generated for this study are included in the manuscript.
